# Utilization of Whole Exome Sequencing Data to Identify Clinically Relevant Pharmacogenomic Variants in Pediatric Inflammatory Bowel Disease

**DOI:** 10.14309/ctg.0000000000000263

**Published:** 2020-12-01

**Authors:** Daniel J. Mulder, Sam Khalouei, Neil Warner, Claudia Gonzaga-Jauregui, Peter C. Church, Thomas D. Walters, Arun K. Ramani, Anne M. Griffiths, Iris Cohn, Aleixo M. Muise

**Affiliations:** 1 Division of Gastroenterology, Hepatology and Nutrition, The Hospital for Sick Children, Toronto, Ontario, Canada;; 2 SickKids Inflammatory Bowel Disease Center and Cell Biology Program, Research Institute, Hospital for Sick Children, Toronto, Ontario, Canada;; 3 Centre for Computational Medicine, The Hospital for Sick Children, Toronto, Ontario, Canada;; 4 Department of Pediatrics and Biochemistry, University of Toronto, Hospital for Sick Children, Toronto, Ontario, Canada;; 5 Regeneron Genetics Center, Regeneron Pharmaceuticals, Tarrytown, New York, USA; and; 6 Department of Clinical Pharmacology and Toxicology, The Hospital for Sick Children, Toronto, Ontario, Canada.

## Abstract

**INTRODUCTION:**

We hypothesized that variants within clinically relevant pharmacogenes could be identified using a whole exome sequencing data set derived from a cohort of more than 1,000 patients with inflammatory bowel disease (IBD).

**METHODS:**

Pediatric patients diagnosed with IBD underwent whole exome sequencing. We selected 18 genes with supporting literature where specific exonic variants would influence clinical care.

**RESULTS:**

We identified actionable pharmacogenomic variants in 63% of patients. Importantly, 5% of patients with IBD were at risk for serious adverse effects from anesthesia and 3% were at increased risk for thrombosis.

**DISCUSSION:**

We identified exonic variants in most of our patients with IBD that directly impact clinical care.

## INTRODUCTION

Pharmacogenomics presents a major opportunity to improve clinical care in a cost-effective manner for patients, especially those with complex diseases who often require a broad range of medications over the course of many years of living with disease. In pediatric inflammatory bowel disease (IBD), whole exome sequencing (WES) enables the genetic diagnosis of monogenic variants in a subset of patients (1). However, the collected WES data are underutilized and may also be used to identify loci or known variants important for drug metabolism and/or increased risk for adverse drug reactions (2,3). We hypothesized that exonic variants within clinically relevant pharmacogenes could be identified in a previously collected WES data set from a single-center cohort of more than 1,000 pediatric patients with IBD (1).

## METHODS

Please see the Supplementary Materials section for detailed methodology.

Patients with IBD younger than 18 years and followed at the Hospital for Sick Children in Toronto had next generation WES, as previously described (1). We identified 18 pharmacogenes (see Supplementary Tables 1, Supplementary Digital Content 1, http://links.lww.com/CTG/A449 and 2) with relevance to patients with IBD (4). Gene selection was based on known pharmacogenomics guidelines including review of the Clinical Pharmacogenomics Implementation Consortium guidelines and those collected in the online Pharmacogenomics Knowledgebase (pharmgkb.org). We limited our search to pharmacogenomic variants that were covered in WES and for which precision therapy is recommended (through avoidance or altered dosing of medications).

Individual WES (n = 1,097, including pediatric patients and affected relatives with IBD) was aligned to the current reference genome (GRCh37) using the Genome Analysis Toolkit framework. Variants were called and annotated using Genome Analysis Toolkit version 3.6. Quality control measures were used to eliminate variant calls of poor quality (see Supplemental Material). Genome mining program GEMINI version 0.18 was used to identify variants of interest. Stargazer version 1.0, which uses the PharmVar database, was used to identify each patient's star haplotype (a predefined pharmacogenomic designation for variant profiles that effect drug metabolism) for the genes of interest.

## RESULTS

**Figure 1.**
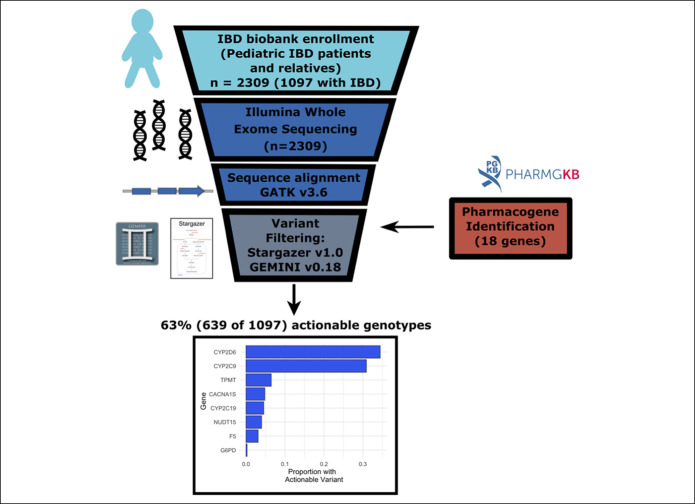
Flowchart of our pharmacogenomic analysis pipeline. After enrolment (n = 2,309), each patient underwent whole exome sequencing and sequence alignment. Available family members were also sequenced. Quality control hard filters were applied prior to variant calling (see Supplementary Methods, http://links.lww.com/CTG/A449). Analyzed samples were limited to patients and family members with IBD (n = 1,097). Pharmacogenes relevant to patients with IBD were identified by literature review and evaluation of pharmGKB (total of 18 genes). Variant filtering was performed using Stargazer and GEMINI frameworks. In our cohort, there were 8 relevant pharmacogenes with variants that would alter clinical care based on current guidelines and standard of care. Sixty-three percent of the patients had at least one variant that could impact care. IBD, inflammatory bowel disease.

Our search strategy (see Supplementary Material, Supplementary Digital Content 1, http://links.lww.com/CTG/A449 and Figure 1) identified relevant exonic variants in 8 of the 18 pharmacogenes examined (Table 1) including clinically relevant variants applicable to IBD treatment with azathioprine in either *TPMT* or *NUDT15* in 10% of our patients. Variants in cytochrome P450 pathway enzymes that are involved in metabolism of adjunct IBD therapies including antidepressant and pain medications were identified in the highest number of patients (58%). We also identified 3 patients with clinically actionable *G6PD* variants. Overall, we identified at least 1 actionable variant in 63% of patients in our cohort, and importantly, 5% of patients with IBD were at risk for serious adverse effects from anesthesia and 3% were at increased risk for thrombosis.

**Table 1.**
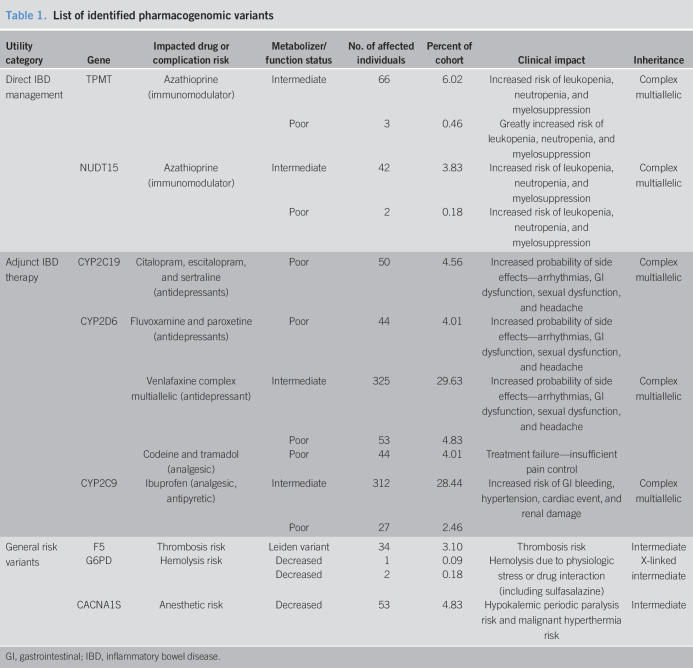
List of identified pharmacogenomic variants

Utility category	Gene	Impacted drug or complication risk	Metabolizer/function status	No. of affected individuals	Percent of cohort	Clinical impact	Inheritance
Direct IBD management	TPMT	Azathioprine (immunomodulator)	Intermediate	66	6.02	Increased risk of leukopenia, neutropenia, and myelosuppression	Complex multiallelic
			Poor	3	0.46	Greatly increased risk of leukopenia, neutropenia, and myelosuppression	
	NUDT15	Azathioprine (immunomodulator)	Intermediate	42	3.83	Increased risk of leukopenia, neutropenia, and myelosuppression	Complex multiallelic
			Poor	2	0.18	Increased risk of leukopenia, neutropenia, and myelosuppression	
Adjunct IBD therapy	CYP2C19	Citalopram, escitalopram, and sertraline (antidepressants)	Poor	50	4.56	Increased probability of side effects—arrhythmias, GI dysfunction, sexual dysfunction, and headache	Complex multiallelic
	CYP2D6	Fluvoxamine and paroxetine (antidepressants)	Poor	44	4.01	Increased probability of side effects—arrhythmias, GI dysfunction, sexual dysfunction, and headache	
		Venlafaxine complex multiallelic (antidepressant)	Intermediate	325	29.63	Increased probability of side effects—arrhythmias, GI dysfunction, sexual dysfunction, and headache	Complex multiallelic
			Poor	53	4.83		
		Codeine and tramadol (analgesic)	Poor	44	4.01	Treatment failure—insufficient pain control	
	CYP2C9	Ibuprofen (analgesic, antipyretic)	Intermediate	312	28.44	Increased risk of GI bleeding, hypertension, cardiac event, and renal damage	Complex multiallelic
			Poor	27	2.46		
General risk variants	F5	Thrombosis risk	Leiden variant	34	3.10	Thrombosis risk	Intermediate
	G6PD	Hemolysis risk	Decreased	1	0.09	Hemolysis due to physiologic stress or drug interaction (including sulfasalazine)	X-linked intermediate
			Decreased	2	0.18		
	CACNA1S	Anesthetic risk	Decreased	53	4.83	Hypokalemic periodic paralysis risk and malignant hyperthermia risk	Intermediate

GI, gastrointestinal; IBD, inflammatory bowel disease.

We did not identify any relevant exonic variants in *HBA1/2* or *HBB* (thalassemia related anemia), *RYR1* (anesthetic risk), or *JAK2* (thrombosis risk) (see Supplemental Table 1, Supplementary Digital Content 1, http://links.lww.com/CTG/A449). The allele frequencies for our cohort were similar to those found in the general population, when we compared our samples with those in the Genome Aggregation Database (gnomAD) (5). We did not include in our final calculations patients with *CYP2D6* variants that result in altered ondansetron/tropisetron metabolism, as the current Clinical Pharmacogenomics Implementation Consortium guideline recommendation is that these patients are not treated differently. Both *ABCB1* and *SLCO1B1* have been identified to contribute to methotrexate toxicity; given that no specific recommendations exist regarding these variants, we did not include them in our total actionable variant count but did include them in Supplemental Table (see Supplementary Digital Content 1, http://links.lww.com/CTG/A449).

## DISCUSSION

We identified established pharmacogenomic variants that are currently recommended to necessitate alterations in pharmacologic or other medical management in most of our patients who underwent WES on a research basis to identify monogenic forms of disease. The findings of clinically actionable variants in such a large portion of our cohort, although expected, is important because it demonstrates that genetics can be better used in clinical care.

Lifelong alterations to treatment based on next generation sequencing (NGS) has wide implications for improving the standard of care for patients with IBD. Currently, WES is most often performed in pediatric patients with IBD to identify rare damaging monogenic variants that are responsible for disease and may inform optimal therapy (1). Increasingly, targeted sequencing panels are being replaced with WES approaches, but with analysis initially limited to a few genes as a more cost effective strategy, a so-called “WES backbone” (6). Leveraging WES data for not only disease diagnosis but also pharmacogenomic applications would be cost effective and improve quality of life for patients with a number of pediatric conditions, including primary immunodeficiency, immune dysregulation, and other gastrointestinal disorders. However, it is worth considering that NGS techniques such as WES and whole genome sequencing (WGS) are not available to all. Targeted sequencing such as *TPMT* genotyping is more broadly used around the world. Future studies should evaluate the cost benefit of broad sequencing in patients with IBD because it can be considered a one-time test that can impact many aspects of care. It is also important to note that it is very likely that the value of NGS for a patient with IBD will increase over time as even more pharmacogenomic variants are linked to clinical care. The estimated one-time cost of WES and even WGS (7) is less than the estimated average cost of the first year of IBD care (8).

The drugs examined here have broad implications because pain medications are commonly used in IBD, and many patients have depression as a comorbidity. Patients with factor V Leiden are at increased risk for thrombotic events and are recommended to avoid estrogen-containing contraception (9). Genomics are also beginning to identify risk alleles potentially influencing response to biologics, including antitumor necrosis factor-α antibodies. The observations of a role for HLADQ haplotype in influencing the likelihood of development of antidrug antibodies could pave the way to personalized decision-making around choice of antitumor necrosis factor and necessity of combination therapy with an immunomodulator (10). However, a limitation of WES compared with WGS is that it does not reliably identify structural variants or noncoding regions; thus, these were not analyzed in our study. For example, the CYP2C19*17 variant allele (rs12248560 (c.-806C>T)) mapping to the promoter region that defines rapid metabolizers of proton pump inhibitors, antidepressants, voriconazole, and other drugs is not captured in standard WES (2,3). Nevertheless, mining WES data for pharmacogenomic variants still had clinical implications for most patients in our cohort, implying that the cost-benefit balance of WES and WGS still requires further consideration.

In conclusion, using exome data derived for other purposes, we identified pharmacogenomic variants in most of our patients that could immediately impact clinical care based on current guidelines. The identification of medically actionable pharmacogenomic variants could have an impact on care and medication prescription for the remainder of the patient's life, especially when identified variants increase risk for serious adverse effects from anesthesia or thrombosis. These findings are not limited to pediatric IBD but would allow precision therapy of many pediatric and adult patients.

## CONFLICTS OF INTEREST

**Guarantor of the article:** Aleixo M. Muise, MD, PhD.

**Specific author contributions:** Iris Cohn, MSc, RPh, and Aleixo M. Muise, MD, PhD, are the cosenior authors. D.J.M., S.K., I.C., and A.M.M.: study concept and design. D.J.M., S.K., P.C.C., T.D.W., A.M.G., and A.M.M.: acquisition of data. All authors: analysis and interpretation of data. D.J.M., S.K., N.W., I.C., and A.M.M.: drafting of the manuscript. All authors: critical revision of the manuscript for important intellectual content.

**Financial support:** A. M. Muise is funded by the Leona M. and Harry B. Helmsley Charitable Trust, a Canada Research Chair (Tier 1) in Pediatric IBD, Canada Institute of Health Research (CIHR) Foundation Grant and NIDDK (RC2DK118640) Grant. A. M. Griffiths hold the Northbridge Financial Corporation Chair in IBD at SickKids Hospital, University of Toronto. D. J. Mulder is funded by a CIHR-Canadian Association of Gastroenterology (CAG) Fellowship.

**Potential competing interests:** C. Gonzaga-Jauregui is a full-time employee of the Regeneron Genetics Center from Regeneron Pharmaceuticals and receives stock options as part of compensation. All other authors declare no competing interests.

**Study Highlights**

**WHAT IS KNOWN**

✓ Patients with inflammatory bowel disease (IBD) require many medications.
✓ Many known genetic variants impact medication effect.

**WHAT IS NEW HERE**

✓ Actionable pharmacogenomic variants were found in most patients with (63%) IBD.
✓ Five percent were at risk for serious adverse effects from anesthesia, and 3% were at increased risk for thrombosis.

**TRANSLATIONAL IMPACT**

✓ Actionable pharmacogenomic variants could have immediate and long-term impact on care for many patients with IBD.

## Supplementary Material

SUPPLEMENTARY MATERIAL
