# Supplementary material for: Utilization of Whole Exome Sequencing Data to Identify Clinically Relevant Pharmacogenomic Variants in Pediatric Inflammatory Bowel Disease
Source: Clin Transl Gastroenterol. 2020 Dec 1;11(12):e00263. doi: 10.14309/ctg.0000000000000263 (PMC7710220; doi:10.14309/ctg.0000000000000263)
Supplement: SUPPLEMENTARY MATERIAL [file ct9-11-e00263-s001.docx]

**Supplemental Table.** Genes Analyzed by GEMINI and Stargazer Pipelines, Impact and Cohort Frequency.

| **Gene** | **Variant Location** | **Clinical Risk (and Guideline Source if applicable)** | **Patients with Variant** | **Total Effected Patients** | **Cohort Allele Frequency** | **gnomAD Allele Frequency (a)** |
| --- | --- | --- | --- | --- | --- | --- |
| JAK1 | 1:64846699 | Possible altered JAK inhibitor altered metabolism | 0 | 0 | 0 | 0.00 |
|  | 1:64846699 |  | 0 | 0 | 0 | Not reported |
|  | 1:64846699 |  | 0 | 0 | 0 | Not reported |
| JAK2 | 9:5073770 | Thrombosis | 0 | 0 | 0 | 0.0003444 |
| JAK3 | 19:17836001 | Possible altered JAK inhibitor altered metabolism | 0 | 0 | 0 | Not reported |
|  | 19:17830610 |  | 0 | 0 | 0 | Not reported |
|  | 19:17838268-17838271 |  | 0 | 0 | 0 | Not reported |
|  | 19:17831775-17831796 |  | 0 | 0 | 0 | Not reported |
|  | 19:17834908 |  | 0 | 0 | 0 | Not reported |
|  | 19:17837171 |  | 0 | 0 | 0 | 0 |
|  | 19:17844244-17844246 |  | 0 | 0 | 0 | Not reported |
|  | 19: 17843086 |  | 0 | 0 | 0 | Not reported |
|  | 19:17839585 |  | 0 | 0 | 0 | 0 |
|  | 19:17837938 |  | 0 | 0 | 0 | 0.000003976 |
|  | 19:17843786 |  | 0 | 0 | 0 | 0.000003981 |
| TYK2 | 19:10353648 | Possible altered JAK inhibitor altered metabolism | 0 | 0 | 0 | Not reported |
|  | 19:10368400-10368406 |  | 0 | 0 | 0 | 0.00004772 |
|  | 19:10361817 |  | 0 | 0 | 0 | 0.000007082 |
|  | 19:10378258 |  | 0 | 0 | 0 | Not reported |
|  | 19:10368060 |  | 0 | 0 | 0 | Not reported |
|  | 19:10358003-10358011 |  | 0 | 0 | 0 | 0.000004 |
| HBA | 16:177012 | Anemia | 0 | 0 | 0 | Not reported |
| HBB | 11:5226943 | Anemia | 1 heterozygous | 1 | 0.00046 | 0.0002334 |
| RYR1 (c) | 19:38451841 | Malignant Hyperthermia | 1 homozygous | 1 | 0.00046 | 0.000007073 |
| ABCB1 | 7:87509328 | Methotrexate toxicity | 4 heterozygous 1 homozygous | 1 | 0.66773 | 0.5105 |
| F5 | 1:169549811 | Thrombosis | 48 heterozygous 1 homozygous | 1 | 0.02 | 0.02 |
| CACNA1S | 1:201065942 | Hypokalemic periodic paralysis and malignant hyperthermia | 86 heterozygous 2 homozygous | 2 | 0.03282 | 0.03681 |
|  | 1:201077916 |  | 1 heterozygous |  | 0.00046 | 0.000007963 |
|  | 1:201077915 |  | 1 heterozygous |  | 0.00046 | 0.00001195 |
| G6PD | X:154532257 | Hemolysis | 1 homozygous | 3 | 0.0005 | 0.0005201 |
|  | X:154532269 |  | 0 |  | 0 | 0.0007759 |
|  | X:154532390 |  | 0 |  | 0 | 0.0001422 |
|  | X:154532411 |  | 0 |  | 0 | 0 |
|  | X:154532676 |  | 0 |  | 0 | 0 |
|  | X:154532698 |  | 0 |  | 0 | 0 |
|  | X:154532701 |  | 0 |  | 0 | 0 |
|  | X:154532766 |  | 0 |  | 0 | 0 |
|  | X:154532990 |  | 0 |  | 0 | 0.0001918 |
|  | X:154533044 |  | 5 heterozygous 2 homozygous |  | 0.0009 | 0.001111 |
|  | X:154533596 |  | 1 heterozygous |  | 0.00091 | 0.0006918 |
|  | X:154534125 |  | 1 heterozygous |  | 0.00091 | 0.000005459 |
|  | X:154534345 |  | 0 |  | 0 | 0.000005464 |
|  | X:154534440 |  | 0 |  | 0 | 0.0001662 |
|  | X:154534495 |  | 0 |  | 0 | 0.000044 |
|  | X:154535205 |  | 0 |  | 0 | 0 |
|  | X:154535261 |  | 0 |  | 0 | 0.000009751 |
|  | X:154535270 |  | 0 |  | 0 | 0.000005453 |
|  | X:154536156 |  | 0 |  | 0 | 0.00001091 |
|  | X:154536168 |  | 0 |  | 0 | 0.0001691 |
|  | X:154546061 |  | 0 |  | 0 | 0.0001417 |
| CYP2C9 | *1/*2, *1/*3 (Intermediate) | Ibuprofen - bleeding and acute kidney injury (CPIC) | 312 | 339 |  |  |
|  | *2/*3, *3/*3 (Poor) | Ibuprofen - bleeding and acute kidney injury (CPIC) | 27 |  |  |  |
| CYP2C19 | *2/*2, *2/*3, *3/*3 (Poor) | Select SSRIs (citalopram, escitalopram, sertraline) - increased probability of side effects - arrhythmias, GI dysfunction, sexual dysfunction, headache (CPIC) | 50 | 50 |  |  |
| CYP2D6 | *3/*4, *4/*4, *5/*5, *5/*6 (Poor) | Fluvoxamine - increased probability of side effects - arrhythmias, GI dysfunction, sexual dysfunction, headache (CPIC) | 44 | 378 |  |  |
|  | *3/*4, *4/*4, *5/*5, *5/*6 (Poor) | Paroxetine - increased probability of side effects - arrhythmias, GI dysfunction, sexual dysfunction, headache (CPIC) | 44 |  |  |  |
|  | (1) Two decreased-activity (*9, *10, *17, *29, *36, *41) alleles, or (2) carrying one active (*1, *2, *33, *35) and one inactive (*3-*8, *11-*16, *19-*21, *38, *40, *42) allele, or (3) carrying one decreased-activity (*9, *10, *17, *29, *36, *41) allele and one inactive (*3-*8, *11-*16, *19-*21, *38, *40, *42) allele (Intermediate) | Venlafaxine - increased probability of side effects - arrhythmias, GI dysfunction, sexual dysfunction, headache (DPWG) | 325 |  |  |  |
|  | Two inactive (*3-*8, *11-*16, *19-*21, *38, *40, *42) alleles (Poor) | Venlafaxine - increased probability of side effects - arrhythmias, GI dysfunction, sexual dysfunction, headache (DPWG) | 53 |  |  |  |
|  | *4/*4, *4/*5, *4/*6, *5/*5 (Poor) | Select Opioids (codeine, tramadol) - increased probability of side effects and toxicity - sedation, decreased respiratory drive, pruritus, withdrawal (CPIC) | 44 |  |  |  |
|  | *4/*10,*4/*41, *5/*9 (Intermediate | Antiemetics (ondansetron, tropisetron) - no predicted effect on drug effect (thus, not in final Table) (CPIC) | 49 | 93 (c) |  |  |
|  | 3/*4,*4/*4, *5/*5, *5/*6 (Poor) | Antiemetics (ondansetron, tropisetron) - no predicted effect on drug effect (thus, not in final Table) (CPIC) | 44 |  |  |  |
| SLCO1B1 | *5/*5, *5/*15, *5/17, *15/*15, *15/*17, *17/*17 (Decreased) | Methotrexate - potential GI upset, myelosupression, kidney injury, mucositis/rash | 28 | 28 |  |  |
| NUDT15 | *1/*2, *1/*3 (Intermediate) | Azathioprine - malignancy, myelosupression, susceptibility to infection, liver injury (CPIC) | 42 | 44 |  |  |
|  | *2/*2, *2/*3, *3/*3 (Poor) |  | 2 |  |  |  |
| TPMT | *1/*2, *1/*3, *1/*4 (Intermediate) | Azathioprine - malignancy, myelosupression, susceptibility to infection, liver injury (CPIC) | 66 | 69 |  |  |
|  | *2/*3, *3/*3, *3/*4 (Poor) |  | 3 |  |  |  |

CPIC, Clinical Pharmacogenetics Implementation Consortium; DPWG, Dutch Pharmacogenetics Working Group

(a) gnomAD v2.1.1

(b) See https://www.pharmgkb.org/guidelineAnnotation/PA166180457 for full list of RYR1 and CACNAS1 variants investigated, this was only positive variant

(c) these individuals are not included in the final unique effected individuals in next column, as variants do not result in altered treatment approach
